# Flavonoid Bioavailability and Attempts for Bioavailability Enhancement

**DOI:** 10.3390/nu5093367

**Published:** 2013-08-28

**Authors:** Surangi H. Thilakarathna, H. P. Vasantha Rupasinghe

**Affiliations:** Department of Environmental Sciences, Faculty of Agriculture, Dalhousie University, P.O. Box 550, Truro, NS B2N 5E3, Canada; E-Mail: thilakarathnas@nsac.ca

**Keywords:** flavonoids, bioavailability, diet, health, disease

## Abstract

Flavonoids are a group of phytochemicals that have shown numerous health effects and have therefore been studied extensively. Of the six common food flavonoid classes, flavonols are distributed ubiquitously among different plant foods whereas appreciable amounts of isoflavones are found in leguminous plant-based foods. Flavonoids have shown promising health promoting effects in human cell culture, experimental animal and human clinical studies. They have shown antioxidant, hypocholesterolemic, anti-inflammatory effects as well as ability to modulate cell signaling and gene expression related disease development. Low bioavailability of flavonoids has been a concern as it can limit or even hinder their health effects. Therefore, attempts to improve their bioavailability in order to improve the efficacy of flavonoids are being studied. Further investigations on bioavailability are warranted as it is a determining factor for flavonoid biological activity.

## 1. Introduction

Consumption of fruits and vegetables has been shown to improve health and directly or indirectly reduce the risk of numerous chronic diseases [1]. Increasing one serving of fruit and vegetable intake per day could reduce the risk of coronary heart disease by 4% [2]. It is believed that the nutrients and non-nutrient bioactive compounds present in fruits and vegetables are responsible for these health benefits. Among numerous plant-based bioactive compounds, flavonoids have been studied extensively and have shown promising results improving various disease aspects. Flavonoids belong to the comprehensive category of polyphenols and six classes, namely flavan-3-ols (including proanthocyanidins), flavonols, anthocyanins, isoflavones, flavanones and flavones are found in plant foods.

Flavonoids exert beneficial effects on health directly and/or indirectly. Previous literature shows evidence that flavonoids (proanthocyanidins as an example) are antioxidants [3] and hypocholesterolemic compounds [4] as well as modulators of cell signaling and gene expression in different experimental models [5] and in human epidemiological studies [6]. Considering the distribution of flavonoids, some classes can be found in many foods whereas the presence of some classes is limited to certain foods. As an example, flavonols can be commonly found in fruits, vegetables and teas; however, isoflavones are almost exclusively located in leguminous plants [7]. Dietary sources of flavonoids and their intake can vary widely among populations as it depends on the availability of dietary sources, dietary practices and food habits of different demographic groups [6,8,9,10,11].

Although flavonoids have shown countless health benefits, their low bioavailability has been a concern. Phase 2 metabolism is known to affect the bioavailability of flavonoids in humans [12]. Usually, most flavonoids undergo sulfation, methylation and glucuronidation in the small intestine and liver [13] and conjugated metabolites can be found in plasma after flavonoid ingestion [14]. In general, metabolites of flavonoids show reduced bioactivity in comparison to parent compounds but there have been results that reported otherwise as well [15]. Despite the bioactivity expressed in different *in vitro* systems, bioavailability of flavonoids would be a determinant factor of their bioactivity *in vivo*. Therefore, enhancement of bioavailability would be of utmost importance in order to exert health effects, *in vivo*. In this regard, numerous attempts have been made to increase bioavailability such as: improving the intestinal absorption via use of absorption enhancers [16], novel delivery systems [17]; improving metabolic stability [18,19]; changing the site of absorption from large intestine to small intestine [20].

This review briefly discusses: flavonoids belonging to the six classes, some of the factors that determine their bioavailability, recent studies attempting to improve their bioavailability that in turn can improve their bioactivity *in vivo*.

## 2. Flavonoids

**Figure 1:**
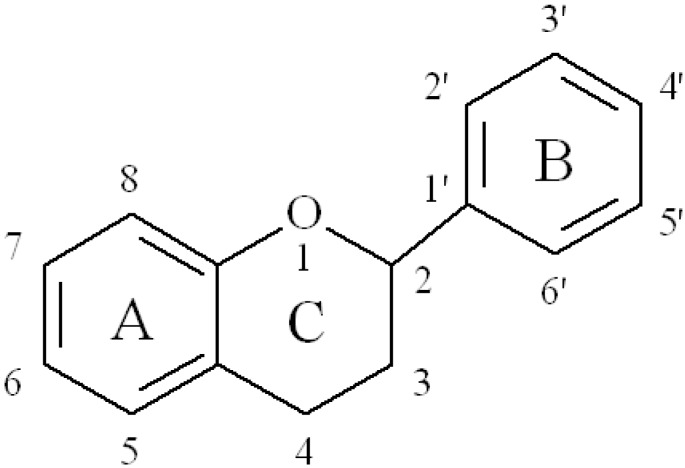
General chemical structure of flavonoids.

Flavonoids are secondary metabolites in plants and fall under the broad category of polyphenols. Scientists have discovered more than 7000 flavonoids and the list of new discovered flavonoids keeps on growing [7]. Flavonoids consist of two aromatic carbon rings connected with a three-carbon bridge (Figure 1). Based on the “C” ring, there are six common food flavonoid classes: flavonols, flavan-3-ols (monomeric and condensed tannins), isoflavones, flavanones, flavones and anthocyanins. Most of the flavonoid compounds except flavan-3-ols (monomers and proanthocyanidins) exist as glycosides [6]. Different flavonoid compounds have shown numerous health promoting effects and have been studied extensively using different experimental model systems. The six classes of flavonoids, their intake and common dietary sources in four different populations are discussed briefly in this section. The common structures and some sources of the six flavonoid classes are shown in Table 1.

**Table 1:** Flavonoid classes, common compounds, their dietary sources and amounts *.

Flavonoid class (Common compounds)		Common food sources and amounts (mg/100 g edible portion)				
**Flavan-3-ols** (**i**) (+)-Catechin, (**ii**) (−)-Epicatechin, (**iii**) Procyanidin B2 (dimer) an example; food values for all dimers present		**Source**		**(i) [21]**	**(ii) [21]**	**(iii) [22]**
		Apples (Red Delicious, with skin) ^1^		2.00	9.83	15.12
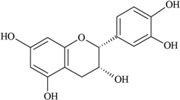	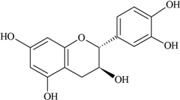	Apricots (raw) ^1^		3.67	4.74	1.33
		Peaches (raw) ^1^		4.92	2.34	12.24
		Pears (raw) ^1^		0.27	3.76	2.73
		Strawberries (raw) ^1^		6.65	1.56	5.26
(**i**)	(**ii**)	Black tea (brewed) ^2^		1.51	2.13	3.74
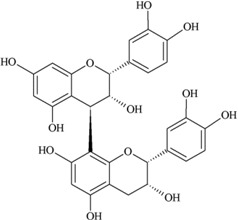		Blueberries (highbush, raw) ^3^		5.29	0.62	5.71
		Cranberries (raw) ^3^		0.39	4.37	25.93
		Cocoa (dry powder) ^4^		64.82	196.43	183.49
		Grapes (black/red) ^5^		0.82	0.96	2.38
		Red wine (table) ^5^		7.12	3.76	20.49
			
(**iii**)						
**Flavonols** (**i**) Kaempferol, (**ii**) Myricetin, (**iii**) Quercetin-3-*O*-glucoside an example of a glucoside, food values are for quercetin		**Source [21]**		**(i)**	**(ii)**	**(iii)**
		Blueberries (highbush) ^3^		1.66	1.26	7.67
		Garlic ^6^		0.26	1.61	1.74
		Onions ^6^		0.63	0.03	21.40
		Kale ^7^		46.80	0.00	22.58
		Broccoli ^7^		7.84	0.06	3.26
		Spinach ^8^		15.75	-	5.75
		Black tea (brewed) ^2^		1.31	0.45	1.99
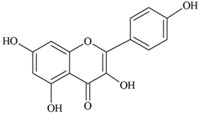	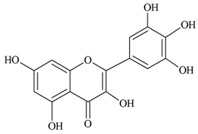	Red wine ^5^		0.20	0.83	1.76
		Cherry tomatoes ^9^		0.10	-	2.76
		Can be found ubiquitous in plant families.				

(**i**)	(**ii**)					
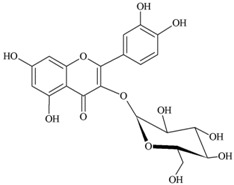						
(**iii**)						
**Anthocyanins** (**i**) Cyanidin-3-*O*-glucoside, (**ii**) Delphinidin-3-*O*-glucoside, (**iii**) Malvidin-3-*O*-glucoside, (**iv**) Pelargonidin-3-*O*-glucoside, Food values are for anthocyanidins (without the sugars)		**Source [21]**	**(i)**	**(ii)**	**(iii)**	**(iv)**
		Apples ^1^	1.27	0.00	0.00	0.00
		Blueberries (lowbush) ^3^	17.92	34.00	54.00	2.65
		Red wine ^5^	0.45	2.75	15.29	-
		Strawberries ^1^	1.63	0.31	0.01	25.69
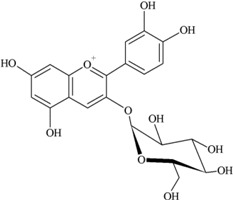	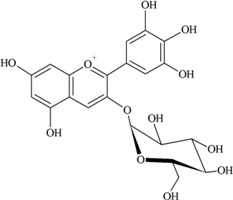	Usually in any pink to purple fruit or vegetable; except the Chenopodiaceae family (beets, quinoa, spinach, Swiss chard, *etc.*).				
				
(**i**)	(**ii**)					
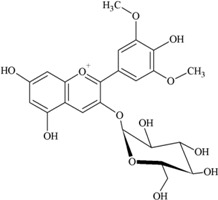	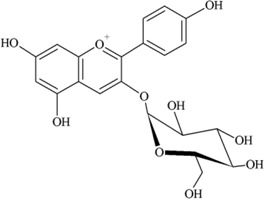					
				
				
				
				
(**iii**)	(**iv**)					
**Isoflavones** (**i**) Daidzein, (**ii**) Genistein, (**iii**) Glycitein		**Source [23]**		**(i)**	**(ii)**	**(iii)**
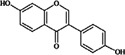	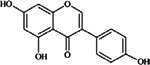	Tofu (regular, raw) ^10^		8.56	12.99	1.98
		Tempeh ^10^		22.66	36.15	3.82
		Soybean (raw, mature seeds, USA) ^10^		61.33	86.33	13.33
(**i**)	(**ii**)	Peanuts (raw, all types) ^10^		0.02	0.24	0.26
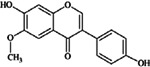		Beans (common, raw) ^10^		0.29	0.30	0.00
		In the Fabaceae (legume) family especially the genus Glycine (soy), but also in small amounts in other plants.				
(**iii**)						
**Flavanones** (**i**) Eriodictyol, (**ii**) Hesperetin, (**iii**) Naringenin		**Source [21]**		**(i)**	**(ii)**	**(iii)**
		Grapefruit (juice, white) ^11^		0.65	2.35	18.23
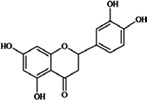	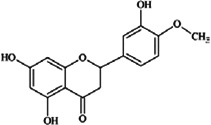	Lemon (juice) ^11^		4.88	14.47	1.38
		Orange (juice) ^11^		0.17	20.39	3.27
		Peppermint ^13^		30.92	9.52	-
			
(**i**)	(**ii**)					
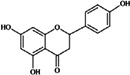						
			
(**iii**)						
**Flavones** (**i**) Apigenin, (**ii**) Luteolin		**Source [21]**		**(i)**	**(ii)**	
		Celery ^13^		2.85	1.05	
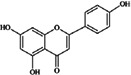	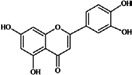	Celery seed (spice) ^13^		83.70	811.41	
		Parsley ^13^		215.46	1.09	
		Green peppers ^9^		0.00	4.71	
		Peppermint ^12^		8.71	11.33	
(**i**)	(**ii**)	Common in leafy plants particularly Apiaceae family.				

* Source: [21,22,23]. Plant families are given in italic superscripts: ^1^ Rosaceae; ^2^ Theaceae; ^3^ Ericaceae; ^4^ Malvaceae; ^5^ Vitaceae; ^6^ Alliaceae; ^7^ Brassicaceae; ^8^ Chenopodiaceae; ^9^ Solanaceae; ^10^ Fabaceae; ^11^ Rutaceae; ^12^ Lamiaceae; ^13^ Apiaceae.

### 2.1. Flavan-3-ols

#### 2.1.1. Monomeric Flavan-3-ols: Catechin and Epicatechin

Flavan-3-ols are monomers and are the units for the oligomers and polymers known as proanthocyanidins or condensed tannins. Some examples of monomeric flavan-3-ols are catechin, epicatechin, gallocatechin, epigallocatechin, epigallocatechin gallate, *etc.* (Table 1). As reviewed by D’Archivio *et al*. [24], catechin and epicatechin are most commonly found in fruits (except citrus) whereas most other monomeric flavan-3-ols are commonly found in different teas (*Camellia sinensis*). Catechin and epicatechin are two flavan-3-ols most commonly found in apple peels [25,26] and about one half of the flavan-3-ols are monomeric in immature apples [27]. These compounds were found in lower concentrations in the flesh than in the skins of fruits [25]. Different types of berries, cherries, grapes, plums, apricots [28,29], red wine, chocolate and teas [24] are good sources.

Consumption of flavan-3-ols can vary among different populations due to different dietary sources. Average daily monomeric catechin and epicatechin intake of adult Australians were 9.36 mg and 16.64 mg respectively and black tea, green tea, apples and wine contributed the most [8]. In a Spanish population, average monomeric catechin and epicatechin intake per day was 13.05 ± 14.71 mg and 11.11 ± 9.63 mg, respectively and similar to the Australian population, wine, tea and apples contributed most to the flavan-3-ol intake [11]. According to a study on middle-aged Japanese women, 98% of the flavan-3-ol intake was from tea followed by apples which contributed to 1.9% of the daily intake [9].

#### 2.1.2. Proanthocyanidins or Condensed Tannins

Proanthocyanidins are dimers, trimers, oligomers (4–10 units) or polymers (>10 units) of monomeric catechin or epicatechin units. Oligomeric and polymeric proanthocyanidin content is difficult to estimate due to their wide range of structures and molecular weights [30]. However, experimental evidence suggests that proanthocyanidins possess high antioxidant activity as these high molecular weight structures can easily complex with metal ions and proteins [31] and antioxidant activity increased with the degree of polymerization for compounds with (−)-epicatechin as the structural unit [31]. Contrastingly, a recent study revealed proanthocyanidins with lower molecular weight were better superoxide and hydroxyl radical scavengers and xanthine oxidase inhibitors [32].

These compounds occur in abundance in grapes, apples, various berries, tea, wine, beer, chocolate [24], *etc*. Immature fruits tend to contain more of these compounds than ripe fruits [33]. Procyanidin content is reported to be significantly higher in unripe apples than in ripe apples [33].

### 2.2. Flavonols

These compounds are nearly ubiquitous in foods [7]. Some food sources are apples, berries, red grapes [28,29], onions, red wine, teas, curly kale, leeks, broccoli, [24], *etc*. Some examples of flavonol compounds are isorhamnetin, kaempferol, myricetin, and quercetin. In apples, flavonols, especially quercetin glycosides are almost exclusively located in the apple peel [25,34]. Flavonols are present mostly in their conjugated forms, and apples contain a mixture of quercetin glycosides and traces of aglycone quercetin [26,27].

Mean quercetin, kaempferol and myricetin daily intake of Australian adults was 12.53 mg, 5.6 mg and 2.4 mg, respectively [8]. Black and green tea contributed most to the intake of the three compounds. Apples were also an important contributor for quercetin intake but not for other compounds. Quercetin and kaempferol contributed more to flavonol intake of Chinese adults [10] and Japanese middle-aged women [9]. In Spanish adults, quercetin accounted for 79.6% of flavonol intake and onions were the main dietary source contributing to 22.6% of the intake followed by lettuce, red wine and apples [11].

### 2.3. Anthocyanins

Anthocyanins are natural pigments in plants and exhibit a blue, purple or red color [24,30]. These compounds are abundant in purple berries, apples, cherries, red and purple grapes and pomegranates [35,36] as well as red wine and certain vegetables such as cabbage, onions and radishes [24]. Common food anthocyanins are cyanidin, delphinidin, malvidin, pelargonidin, peonidin and petunidin.

According to Somerset and Johannot [8], major dietary sources of anthocyanins in adult Australians were blueberries, cherries and wine. Wine contributed around 85%–95% of the daily intake of delphinidin, petunidin and malvidin [8]. In Spanish adults, red wine was the main contributor to anthocyanin intake (45.62%) and cyanidin (35.74%) and malvidin (35.99%) were the main compounds [11].

### 2.4. Isoflavones

Isoflavones are primarily found in leguminous plants and specifically soy and its products are excellent sources [7,35]. Compounds that belong to this class of flavonoids include biochanin A, daidzein, genistein, and glycitein. Isoflavones have phytoestrogenic activity and can bind to estrogen receptors [24]. Tofu (42.2%), natto (28.7%) and miso (16.3%) contributed more to the average daily isoflavone intake of middle-aged Japanese women [9] while non-fermented legume foods like soybean sprouts (71.96%), beans (17.46%) and chickpeas (7.96%) contributed more to the intake of Spanish adults [11]. Clearly from the above information, food habits have played a role in the isoflavone intake where Japanese populations consume more soy products (mostly fermented) and legumes and Spanish populations consume less.

### 2.5. Flavanones

Some examples of flavanones are eriodictyol, hesperetin, and naringenin. These compounds are commonly found in citrus fruits and to a lesser extent in tomatoes and mint [24,35]. Total hesperetin, naringenin and eriodictyol intake per day by Australian adults was 4.2 mg, 2.1 mg and 0.5 mg, respectively [8]. Comparatively, the daily intake of these three compounds were considerably higher in the Spanish adults where hesperetin, naringenin and eriodictyol daily intakes were 31.26 ± 29.85 mg, 18.29 ± 16.84 mg and 1.00 ± 1.36 mg, respectively [11]. Interestingly, the main dietary source for both populations was oranges.

### 2.6. Flavones

Flavones are the less common flavonoids [24] and can be found mainly in celery, green leafy herbs like parsley and chamomile [7,35,37]. Apigenin and luteolin are two compounds that belong to this class. Daily average apigenin and luteolin intake by adult Australians were 0.45 mg and 0.08 mg, respectively; parsley and celery were the main dietary sources of apigenin where, celery and English spinach were the main dietary sources of luteolin [8]. The daily average dietary intake of these two flavones was comparatively higher in Chinese adults. According to the data reported by Zhang *et al*. [10], daily average apigenin and luteolin intake of Chinese adults were 1.06 ± 0.56 mg and 3.82 ± 1.88 mg, respectively and their major dietary sources were eggplant and celery, respectively. In middle-aged Japanese women, Malabar spinach and green pepper contributed to 91.8% of the daily average flavone intake and parsley and celery contributed less than 4% [9]. Interestingly, red wine (32.18%) and oranges (24.25%) contributed more to the flavone intake of Spanish adults and the average daily flavone intake was 3.4 ± 2.92 mg [11].

## 3. Bioavailability of Dietary Flavonoids

### 3.1. Metabolism and Bioavailability

**Figure 2:**
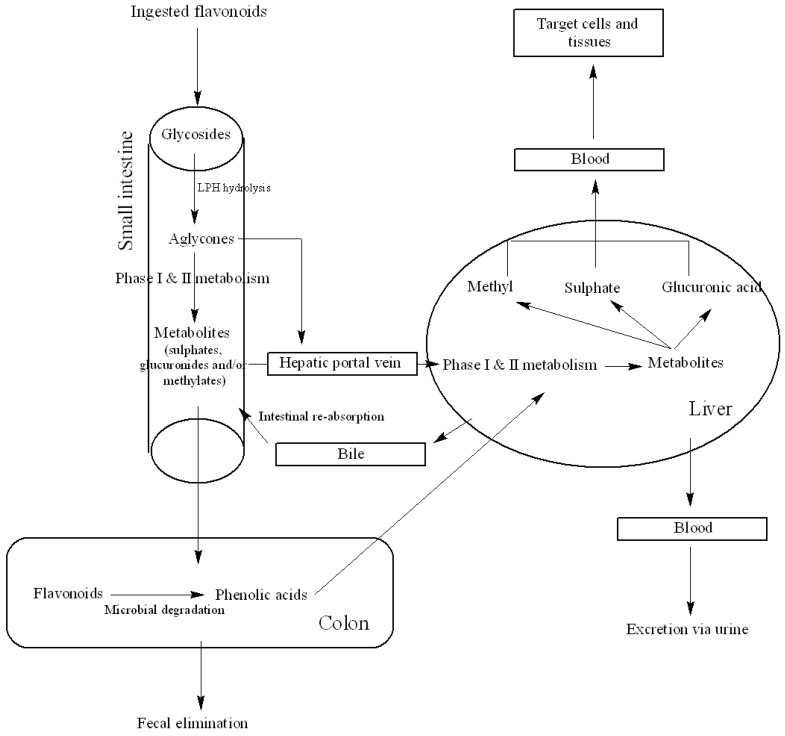
A simplified schematic of human flavonoid metabolism. Ingested flavonoids undergo extensive intestinal metabolism. Metabolites are then transported to the liver via hepatic portal vein and undergo further metabolism. The liver metabolites can be transported to targeted cells and tissues, excreted to bile and undergo enterohepatic re-circulation, or eliminated via urine and/or feces. The aglycones or flavonoid metabolites that reach the colon can undergo microbial degradation and reabsorption (LPH: lactase-phlorizin hydrolase; adapted from [43] with modifications).

According to U.S. Food and Drug Administration (FDA), the definition of bioavailability is “the rate and extent to which the active ingredient or active moiety is absorbed from a drug product and becomes available at the site of action” [38]. This same principle can be applied to flavonoid compounds in food. As the definition implies, the rate of absorption and the availability at the site of action is of utmost importance for a bioactive to be effective within biological systems and thus be “bioavailable”. Therefore, it is imperative to determine the amount of a specific nutrient or bioactive compound in a food or dietary supplement as well as its bioavailability. Despite the health claims of flavonoids, it is a known fact that the bioavailability of flavonoids is generally low and can vary drastically among different flavonoid classes as well as individual compounds in a particular class. Relative urinary excretion of anthocyanins and daidzin intake was 0.3% and 43% respectively which explains the variability in bioavailability of flavonoids [39]. When it comes to flavonoids with complex structures and larger molecular weights, bioavailability may be even lower [39,40]. (The content discussed under this and the following section is summarized in Table 2). Metabolism of flavonoids in general is illustrated in Figure 2. Flavonoids are substrates for conjugating and hydrolyzing enzymes in the small intestine, liver and colon and all are conjugated to *O*-glucuronides, sulfate esters and *O*-methyl esters and hardly any aglycones are present in the plasma [39]. Conjugation of flavonoids first occurs in the small intestine followed by the liver where they are further metabolized and the produced glucuronides and sulfate derivatives facilitate their excretion via urine and bile [39]. The compounds that are not absorbed in the intestine will reach the colon and be subjected to structural modifications by colonic microflora [41]. The flavonoid glucuronides that re-enter the enterohepatic circulation through bile excretion are hydrolyzed by the microbiota to aglycones [39,40]. Aglycones can further be catabolized to low molecular weight compounds that can readily be absorbed. The following paragraphs will discuss the bioavailability of compounds belonging to different flavonoid classes in brief. 

As reviewed by Spencer *et al*. [42], monomeric flavan-3-ols are extensively metabolized to *O*-methylated forms and/or conjugated to glucuronides or sulphates during systemic absorption. Further, after reviewing different studies, the major bioactive forms of the monomeric flavan-3-ols as well as procyanidins are most likely metabolized to conjugates of epicatechin [42]. A study carried out on the absorption and metabolism of flavan-3-ols in humans after consuming a ready-to-drink tea (iced tea), confirmed the above statements where eight metabolites and conjugates of epicatechin were excreted in urine [41]. According to them, the major urinary excreted flavan-3-ol metabolites from iced tea were epigallocatechin-*O*-glucuronide and methyl-epicatechin-sulphate.

Metabolism, rate and amount of dietary quercetin absorption in humans appear to depend primarily on the type and position of sugar moiety [44]. According to Graefe *et al*. [44], bioavailability of quercetin metabolites were five times higher when quercetin was administered as quercetin-4′-*O*-glucoside (C_max_: 2.1 ± 1.6 µg/mL; *t*_max_: 0.7 ± 0.3 h) instead of quercetin-3-*O*-rutinoside (C_max_: 0.3 ± 0.3 µg/mL; *t*_max_: 7.0 ± 2.9 h) and the site of absorption for these glycosides were different. Regardless of the type of quercetin glycoside administered, the potentially active quercetin metabolites (glucuronides) were identical in human plasma. Bioavailability of the ingested quercetin also depends on its dietary source. In a clinical study, consumption of onion powder (C_max_: 273.2 ± 93.7 ng/mL; *t*_max_: 2.0 ± 1.7 h; *t*_1/2_: 14.8 ± 4.8 h) led to faster absorption, higher plasma concentrations and greater bioavailability compared to apple peel powder (C_max_: 63.8 ± 22.4 ng/mL; *t*_max_: 2.9 ± 2.0 h; *t*_1/2_: 65.4 ± 80.0 h) [45]. Quercetin glycosides in onion powder were mainly quercetin-3,4′-*O*-glucoside and 4′-*O*-glucoside whereas in apple peels were quercetin-3-*O*-arabinoside, 3-*O*-galactoside, 3-*O*-glucoside and 3-*O*-rhamnoside [34].

**Figure 3:**
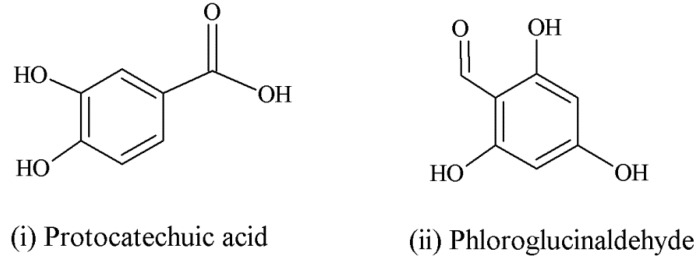
Degradation products of anthocyanins. (i) Protocatechuic acid; (ii) Phloroglucinaldehyde.

It was reported that a significant proportion of intestinal metabolites formed from ingested anthocyanins were likely to be conjugates of protocatechuic acid and phloroglucinaldehyde (Figure 3) [46]. These compounds are the degradation products of anthocyanins. After a 4 h incubation of cyanidin and cyanidin-3-*O*-glucoside in a cell-free culture media, 96% of the cyanidin and 57% of the cyanidin-3-*O*-glucoside were degraded. In cultured human intestinal Caco-2 cell media, these degradation products (protocatechuic acid and phloroglucinaldehyde) were metabolized into their glucuronide and sulfate conjugates [46]. Degradation products and their metabolites are therefore part of anthocyanin bioactivity.

Another recent study carried out on the bioavailability of an anthocyanin derivative, tried to address an aspect in anthocyanin metabolism that might have been overlooked. Fernandes *et al*. [47] studied the bioavailability of an anthocyanin derivative: flavan-3-ol-anthocyanin dimer [(+)-catechin-4,8-malvidin-3-glucoside], using Caco-2 cells to assess the transepithelial transport with comparison to (+)-catechin, malvidin-3-glucoside and procyanidin B3 (dimer). All the tested compounds crossed the Caco-2 cell model barrier and the catechin-malvidin dimer showed significantly less absorption efficiency compared to catechin or malvidin. Transport efficiency of the catechin-malvidin dimer was higher compared to procyanidin B3 dimer; the presence of the anthocyanin and its glucose moiety in the flavan-3-ol-anthocyanin dimer was thought to be responsible for this increased efficiency. Interestingly, no metabolites of the catechin-malvidin dimer were detected after 2 h incubation and only the breakdown of parent compounds was detected. Findings of this recent study suggested that the absorption of anthocyanins can take place as anthocyanin derivatives and certain derivatives, *i.e.*, the catechin-malvidin dimer, are metabolically more resistant than their parent compounds.

A study carried out on the bioavailability of genistein (aglycone) and its glycoside genistin, confirmed that the bioavailability of the aglycone was higher compared to its glycoside form [48]. Major amounts of genistein and its metabolites were recovered from the small intestinal luminal contents and feces. The experiment was carried out on unanesthetized rats and after ingestion of the compounds, blood was collected using a permanently inserted cannula into the portal vein. The isoflavone profile from the portal vein plasma provided an insight into deglycosylation of isoflavones in the small intestine before undergoing hepatic metabolism.

Oral administration of citrus flavanone aglycones, hesperetin and naringenin to human subjects showed a rapid absorption but low bioavailability according to cumulative urinary recovery data [49]. Compounds appeared to undergo extensive first-pass metabolism partly by intestinal bacteria and degraded into phenolic compounds. Hesperetin and naringenin concentrations were observed in plasma 20 min after dosing and reached a peak in 4.0 and 3.5 h, respectively. C_max_ for hesperetin and naringenin were 825.78 ± 410.63 and 2009.51 ± 770.82 ng/mL, respectively.

### 3.2. Factors Affecting Bioavailability

Numerous factors affect the bioavailability of ingested dietary flavonoids. Some factors are discussed briefly under this section. When it comes to flavonoid absorption, chemical structure in terms of molecular weight, glycosylation and esterification play a pivotal role [40].

#### 3.2.1. Molecular Weight

Molecular weight is an important factor that greatly affects the absorption and thus bioavailability of certain flavonoids. One example would be the polymeric proanthocyanidins for which, their larger molecular weight, absorption would practically be impossible [40]. The unaltered proanthocyanidins will reach the colon, get catabolized by microorganisms and the resulting microbial metabolites be absorbed into the circulatory systems and excreted via urine [50]. Colonic breakdown products of proanthocyanidins were suggested to be the actual active compounds that exert biological activity. Contrastingly, Shoji and coworkers [51] concluded that oligomeric apple proanthocyanidins (dimer to pentamer) were absorbed in the digestive tract of rats and free procyanidins were present in their plasma. As reported, free proanthocyanidin levels in rat plasma peeked 2 h after administration and decreased subsequently up to 24 h. Furthermore, proanthocyanidin concentration took much longer to peek when compared to other flavan-3-ols and polyphenols. Therefore, oligomeric procyanidins were assumed to be absorbed in the small intestine and were not degraded to lower molecular weight compounds other than the intact procyanidins 2 h after administration [51]. On the other hand, *in vitro* results have shown that polymeric proanthocyanidins were degraded by the colonic microflora into lower molecular weight compounds and these results have yet to be confirmed *in vivo*.

#### 3.2.2. Glycosylation

Absorption of flavonoids can vary drastically among different classes as well as among different conjugates of the same compound in a particular class. Absorption of some flavonoid glycosides can be very rapid, some can be very slow, and thus affect the bioavailability. Bioavailability of apple quercetin glycosides was 30% of that from onions [52]. The associated sugar moiety had a great influence on absorption; quercetin glucosides was absorbed 10 times faster and the plasma concentration peaked 20 times higher than quercetin rutinosides in humans [53]. It was suggested that glucosides were absorbed in the small intestine whereas quercetin rutinosides may be absorbed in the colon after deglycosylation. The daily plasma level of quercetin was predicted to be substantially less than 1 µM [54]. Catechins are rapidly absorbed and thus the small intestine is the probable site of absorption. The type of catechin monomer was not a factor determining absorption but dimerization reduced bioavailability [54]. Although anthocyanins are rapidly absorbed, their bioavailability was the lowest compared to other flavonoids [54]. These compounds are not easily hydrolyzed to their aglycones [54]. Although most glycosylated flavonoids need to be hydrolyzed to their aglycones for absorption, anthocyanins were absorbed and detected in the circulation without sugars or with their sugars intact [55].

#### 3.2.3. Metabolic Conversion

Once ingested, the flavonoids interact with human conjugating enzymes in the enterocytes as well as in the liver. Metabolic conversion is known as a major factor affecting flavonoid bioavailability. Quercetin aglycone or its glycosides are generally not found in plasma but its glucuronic acid, sulfate or methyl conjugates were exclusively present in plasma [56,57]. Sulfation, methylation and glucuronidation occur in the enterocytes and liver [13] and, generally, most of the flavonoids undergo these metabolic conversions. Contradictory reports have been published on the bioactivity of flavonoid metabolites. Quercetin-3-*O*-glucuronic acid, quercetin-3-sulphate and isorhamnetin-3-glucuronic acid demonstrated LDL oxidation inhibition *in vitro* at physiological concentrations (100 nM) that was not different from the activity of their parent compound, quercetin or its glycosides [15]. Catechin is found exclusively in the plasma as methyl, sulfate and glucuronic acid conjugates and generally has a shorter half-life [12]. Epicatechin was metabolized mainly to sulfate conjugates and not glucuronidated by the liver, small intestine or large intestine [25].

#### 3.2.4. Interaction with Colonic Microflora

Interaction of the flavonoid compounds with colonic microflora was reported to influence their bioavailability. As reviewed by Del Rio *et al*. [42], a significant amount of the ingested flavonoids were not absorbed in the small intestine. They reached the large intestine and were degraded by colonic microflora into simple phenolic acids that were absorbed into the circulatory system [58]. These colonic catabolites were known to express biological activities and potential health properties relevant to flavonoids, although the ingested flavonoids primarily were not absorbed in the small intestine. Proanthocyanidins were known to be catabolized to phenylacetic acid, mono- and dihydroxyphenylacetic acids, mono- and dihydroxyphenylpropionic acids, and hydroxybenzoic acid; anthocyanins were catabolized mainly to protocatechuic acid [58]. As reviewed by Williamson and Clifford [50], a key step in the catabolism of flavonoids is the fission of the A-ring and loss of carbon C_5_ to C_8_ as oxaloacetate which is eventually metabolized to carbon dioxide.

After consuming orange juice containing 168 µM of hesperetin-7-*O*-rutinoside, two hesperetin-*O*-glucuronides appeared in the human plasma with a peak concentration of 922 nmol/L at 4.4 h [59]. Cleavage of the rutinose moiety was suggested to occur due to enzymes of the colonic microflora and was indicative of absorption in the large intestine rather than small intestine [59]. Interesting findings were reported in a study where tomato juice containing rutin (quercetin-3-*O*-rutinoside) was consumed by healthy volunteers and subjects with an ileostomy [60]. In healthy subjects, low concentrations of quercetin-3-*O*-glucuronide and isorhamnetin-3-*O*-glucuronide appeared in the plasma 4 h after ingestion and nine metabolites appeared in urine. In ileostomists, no metabolites were detected in the plasma or urine and around 86% of the rutin was recovered in the ileal fluid. Further, phenolic acid catabolites of rutin appeared in the urine of healthy subjects which accounted for 22% of the rutin intake.

Metabolism of isoflavones is a complicated process which involves the gut microflora. Regarding metabolism, the rate of conjugation is known to be high and therefore, isoflavone aglycones are scarcely present in the circulation after ingestion of isoflavones [61]. Equol is a metabolite formed specifically after daidzein consumption and production of equol from diadzein is greatly influenced by the gut microflora [61,62]. This process also shows a greater inter-individual variation owing to the compositional variation in the gut microflora among different individuals [63,64]. Interestingly, all adult humans cannot produce equol and thus, two subpopulations can be identified based on equol production. The inability to produce equol could be due to the absence of bacterial species that involve in equol production or lack of the appropriate enzyme in the gut microflora [62]. Equol can be responsible for the clinical effectiveness of isoflavones as its bioactivity was reported to be stronger than daidzein [65].

### 3.3. Efforts to Improve Bioavailability

Dietary flavonoids have shown promising anti-atherosclerotic effects in different experimental models (*in vitro*, *ex vivo* and *in vivo*) but contradictory results are often reported in human clinical trials. Low bioavailability of these bioactives in humans plays a key role in these findings. To improve the bioavailability of a bioactive, one has to have a thorough understanding of the metabolism of the bioactive(s) of concern. Some metabolic process areas concerned with improving bioavailability are: increasing the intestinal absorption [16], improving metabolic stability [18,19], changing the site of absorption (from colon to small intestine) [20], *etc*. To achieve these goals microencapsulation, nano-delivery systems, microemulsions, enzymatic methylation of flavonoids were among some of the techniques explored. Under this section, some of the methods used to improve the bioavailability of selected flavonoid compounds are discussed briefly.

#### 3.3.1. Improving the Intestinal Absorption

Borneol/methanol eutectic mixtures have been used as intestinal absorption enhancers of drugs and a similar mixture was used to improve daidzein absorption in a recent study. The borneol/methanol mixture enhanced bioavailability of daidzein by improving its solubility and permeability [16]. The combination of borneol/methanol eutectic mixture with a micro-emulsion, further improved bioavailability of daidzein. Micro-emulsions are lipid-based delivery systems that have greater solubilization, thermodynamic stability and permeability enhancement induced by a surfactant [66]. The micro-emulsion used in the study contained a surfactant (Cremophor RH40, PEG400) and acted as an absorption promoter of the water insoluble daidzein [16]. The study reported similar findings for *in vitro* and rat experiments.

**Table 2:** Summary of the metabolism and bioavailability of flavonoid classes discussed.

Flavonoid class	Molecular Weight	Glycosylation	Metabolic conversion	Colonic microflora
General	Decreases bioavailability	Generally removed	Major factor in bioavailability; can take place in small intestine, liver and colon; usually to glucuronides but also sulphation and methylation [39]; facilitates urinary and biliary excretion [39].	Influence availability; catabolize compounds to low molecular weight compounds that are readily absorbed [58].
Flavan-3-ols (monomeric)			Major bioactive forms: conjugates of epicatechin [41]; catechin: methyl, sulfate and glucuronic acid conjugates; epicatechin: mainly to sulfate conjugates, no glucuronidation [41].	
Proanthocyanidins	Decreases bioavailability [40].		Major bioactive forms: conjugates of epicatechin [41]; oligomeric procyanidins can absorb in small intestine [51].	Influences polymeric proanthocyanidin degradation [50].
Flavonols		Sugars and their position affects bioavailability [44].	Potentially active metabolites: glucuronides [44].	Facilitates glucuronidation [60].
Anthocyanins	Anthocyanin derivatives (flavan-3-ol-anthocyanin dimer) can potentially be absorbed with less efficiency [47].	Sometimes found with sugars intact in circulation [55].	Major intestinal metabolites: glucuronide and sulfate conjugates of protocatechuic acid and phloroglucinaldehyde [46]; anthocyanin derivatives metabolically more resistant than parent compounds [47].	
Isoflavones		Aglycone more bioavailable; possible deglycosylation prior hepatic metabolism [48].		Metabolize daidzein to equol [58].
Flavanones		Rapid absorption, low bioavailability [49].		Extensive first-pass metabolism partly by intestinal bacteria degraded into phenolic compounds [49].

Solid dispersion of daidzein at different daidzein polyvinylpyrrolidone ratios was tested in order to improve aqueous solubility and bioavailability of daidzein [67]. Daidzein solubility in the solid dispersion was eight times more than the free drug in water. Authors stated that the rate-limiting step in daidzein absorption may be the dissolution process and using polyvinylpyrrolidone dispersion as an oral preparation can improve the bioavailability of daidzein.

A nano-delivery system was designed and used to improve the oral bioavailability and intestinal absorption of daidzein. A daidzein-lecithin complex that self-assembled to form micelles with lecithin and sodium bile (nanometer sized particles) significantly improved intestinal absorption [17]. This particular daidzein-lecithin complex had stability during assembly of the micelles and the micelles themselves had good stability over time. In the pharmacokinetic study, the daidzein-lecithin self-assembled micelles distributed mainly in the stomach and proximal intestine after oral administration to rats and the intestinal bioavailability significantly improved compared to the free daidzein suspension [17].

Absorption of an isoflavone extract was improved by complexing with β-cyclodextrin in an *in vitro* system [68]. The aqueous solubility was reported to be 26 times greater than the solubility of the isoflavone extract itself. Oral administration of the extract complexed with β-cyclodextrin (isoflavone glycoside basis) to Sprague-Dawley rats showed enhanced bioavailability of daidzein, genistin and glycitin.

#### 3.3.2. Changing the Site of Absorption

Another approach to improve bioavailability of dietary flavonoids would be to change the site of absorption from large intestine to small intestine. As discussed before, most of the flavonoids pass through the small intestine, reach the colon, and get extensively metabolized into phenolic acids by the colonic microflora. In the hesperidin molecule, rutinose (6-o-α-l-rhamnosyl-d-glucose) is attached at the 7th position of the A ring and are known to be a determinant factor for absorption as glycosides with rhamnose were poorly absorbed compared to hesperetin or hesperetin glucoside [20]. Enzymatic conversion of hesperidin into hesperetin-7-glucoside in orange juice using hesperidinase, improved plasma bioavailability of total hesperetin and reduced the time taken to reach maximum plasma concentration in human subjects when compared to subjects consuming untreated orange juice. The cleavage of the rhamnose moiety changed the absorption site and as a result, bioavailability was improved.

#### 3.3.3. Improving the Metabolic Stability

Blocking the free hydroxyl groups in flavones by capping with methyl groups was tested as an approach to prevent conjugation and improve metabolic resistance and thereby, bioavailability [18]. The two methylated-flavones, 5,7-dimethoxyflavone and 3′,4′-dimethoxyflavone were metabolically very stable compared to a non-methylated form galangin (3,5,7-trihydroxyflavone) which was easily glucuronidated in the liver S9 fraction [69]. As reviewed by Walle [18], this approach greatly improved metabolic stability of flavones *in vitro* and improved bioavailability and tissue distribution of methylated flavones in rats. Genistein and kaempferol along with their monomethylated forms (biochanin A and kaempferide, respectively) were examined for their affinity to human serum albumin and ovalbumin [19]. Serum albumins are the main transport proteins for many xenobiotics. Methylation of flavonoids improved affinity to these proteins by 2–16 times and improved transporting ability. Improved hydrophobicity of flavonoids may be responsible for increasing their binding ability [19].

As an attempt to increase the circulatory and organ levels of quercetin, several ester derivatives of quercetin were formulated to bypass the phase II metabolism during absorption [70]. Quercetin ester derivatives were synthesized and transepithelial transport across monolayers of three cell lines (canine MDCK-1, -2 and human Caco-2) were tested. Certain esters crossed some monolayers (MDCK and some Caco-2 clones) without phase II metabolism whereas for certain Caco-2 clones, complete deacylation occurred followed by metabolism of quercetin. Since elimination of residual acyl groups are expected *in vivo*, this method may increase the systemic levels of quercetin.

#### 3.3.4. Effect of the Food Matrix

Another approach to improve bioavailability of bioactive flavonoids is food ingredient supplementation. For this attempt to be successful, different food ingredients have to be tested with the particular bioactive/s of concern. Bioavailability of green tea catechins were significantly improved in mice by supplementing with steamed rice [71]. Steamed rice supplementation increased the bioavailability of non-gallated catechins, especially epigallocatechin. The authors predicted proline-rich proteins in the rice endosperm are capable of binding gallated catechins like epigallocatechingallate and convert them into non-gallated catechins like epigallocatechin by tannase, thus enhancing epigallocatechin bioavailability.

Carbohydrate composition of the food matrix was suggested to be an important determinant of the total flavonol absorption in the small intestine [72]. Consumption of flavonols along with maltitol decreased the flavonol absorption in the small intestine compared to flavonol consumption with sucrose without changing the catechol-*O*-methyltransferase activity. Furthermore, when flavan-3-ol content was low, influence of the food matrix was greater.

In a randomized, single-blinded, diet-controlled, cross-over study, six healthy females (aged 22–28) consumed 130 mg quercetin equivalents from either quercetin-enriched cereal bars or quercetin powder filled capsules. Systemic availability in terms of plasma concentration-time curves was five times higher and the maximum concentration was six times higher after quercetin-enriched cereal bar ingestion [73].

## 4. Conclusions

In conclusion, though flavonoids have shown promising health properties under experimental conditions, low bioavailability of some flavonoids needs to be enhanced for full exploitation of their therapeutic benefits in prevention and treatment of diseases. Therefore, continuing investigation is required to enhance the bioavailability and subsequent efficacy of certain flavonoids using consumer-friendly technologies.
